# Metabolomics Analysis as a Tool in Periodontitis Diagnosis: A Systematic Review

**DOI:** 10.1002/cre2.70095

**Published:** 2025-04-02

**Authors:** Ana‐Maria Condor, Andreea Iuliana Kui, Smaranda Dana Buduru, Marius Negucioiu, Daniela Cornelia Condor, Patricia‐Ondine Lucaciu

**Affiliations:** ^1^ Department of Oral Rehabilitation, Faculty of Dental Medicine Oral Health Discipline Cluj‐Napoca Romania; ^2^ Cluj County Emergency Clinical Hospital Cluj‐Napoca Romania; ^3^ Department of Prosthodontics and Dental Materials, Faculty of Dental Medicine Prosthodontics Discipline Cluj‐Napoca Romania; ^4^ Department of Oral Rehabilitation, Faculty of Dental Medicine Periodontology Discipline Cluj‐Napoca Romania

**Keywords:** biomarkers, metabolomics, oral health, periodontitis

## Abstract

**Objectives:**

This study aims to summarize recent studies available on untargeted metabolomics employed for periodontitis diagnosis, from saliva and gingival crevicular fluid samples, to identify recurring metabolites with biomarker‐value potential. A secondary objective was to analysudurue the protocols of existing studies, to facilitate further research.

**Material and Methods:**

Three databases were electronically searched for relevant studies (PubMed, Web of Science, Scopus). Risk of bias assessment was performed using the Newcastle‐Ottawa scale (NOS). Data was extracted from studies, regarding general characteristics and conclusions, population characteristics, periodontal protocols, and metabolomics protocols. Metabolic pathway analysis was performed for recurrent metabolites.

**Results:**

After screening 405 studies, 13 studies (10 using saliva samples, 3 using GCF samples) were included. 22 metabolites were identified in more than one study and included into the pathway analysis. Butyrate, lactate, isoleucine, glucose, pyruvate, isovalerate, hypoxanthine/xanthine, proline, valine, phenylalanine, and ethanol were most frequently encountered and were found upregulated in periodontitis patients compared to periodontally healthy patients.

**Conclusions:**

Metabolomics could provide valuable opportunities in validating potential biomarkers or diagnosis panels, contributing to the screening, prognosis, progression and monitoring of periodontitis. Further studies on larger populations and using established protocols are needed. (PROSPERO CRD42023470339).

## 1 Introduction

Periodontitis represents a global oral and public health challenge, being treatable but incurable (Sanz, Herrera, et al. 2020; Papapanou et al. 2018; Peres et al. 2019; Bernabe et al. 2020). Early stages of disease are nearly asymptomatic; while easily detectable for a dental professional by probing and periodontal index applications, the patient or other medical professionals may be unaware of its existence, due to no specific symptoms being present (excepting gingival inflammation and/or bleeding). Thus, patients usually seek medical attention when irreversible tissular support changes are present (Mohd‐Said et al. 2022; Nazir et al. 2020). Untreated, periodontitis leads to masticatory and esthetic impairment, disability, low self‐esteem, and reduced quality of life (Romandini et al. 2021; Ferreira et al. 2017; Uy et al. 2022). In 2021, over 1 billion people were affected by severe periodontitis, demonstrating a severe disease burden (Nascimento et al. 2024). Periodontitis is associated with chronic non‐communicable Diseases (NCDs) and increased mortality risk, through common chronic inflammatory pathways, based on systemic bacterial dissemination, cytokine storms and aberrant host response (Romandini et al. 2021; Sanz, Marco del Castillo, et al. 2020; Wu et al. 2020; Jin et al. 2016; Kim et al. 2022; Ramadan et al. 2020; Hajishengallis and Chavakis 2021). Current diagnosis criteria for periodontitis are based on clinical assessments (Papapanou et al. 2018; Caton et al. 2018; Tonetti et al. 2018). The challenges of treating periodontitis, its prevalence and rising incidence highlight the need for periodontal screening strategies and novel approaches in periodontal diagnosis. Early detection plays a fundamental role in successful treatment and developing screening strategies based on biomarkers could facilitate a favorable prognosis of periodontitis, contributing to better prophylaxis and treatment strategies (Baima, Corana et al. 2021).

Metabolomics directly reflects the state of cells or tissues and their biochemical activity (Klassen et al. 2017). It is an emerging tool in precision medicine, clinical diagnostics and biomarker discovery, which can discern between healthy and periodontally affected individuals (Patti et al. 2012; Clish 2015; Barnes et al. 2011; Alamri et al. 2023; Shi et al. 2020). Metabolomics follows two main approaches: targeted and untargeted. While targeted metabolomics focuses on quantifying specific groups of metabolites (like amino acids, fatty acids, sugars, or lipids) to validate pre‐identified potential metabolic biomarkers or to investigate specific metabolic pathways, untargeted metabolomics involves identifying and quantifying all existing metabolites in a sample, even previously unidentified ones. This approach is better suited for biomarker detection since it involves a global profiling of the metabolome (Zhang et al. 2016). Metabolic biomarkers associated with periodontitis onset and progression could be employed for disease detection, progression monitoring and pathology pathways (Brito et al. 2022). Saliva and GCF are accessible, information‐rich biofluids, which can be sampled quickly and non‐invasively, considered ideal for screening tests (Baima, Corana, et al. 2021; Javaid et al. 2016; Gardner et al. 2020; Baima, Iaderosa, et al. 2021; Khurshid et al. 2021). Several biomarkers for periodontitis have been diagnosed from these biofluids, such as metalloproteinase‐8 (MMP‐8), interleukin 1‐beta and interleukin 6 (IL‐1 β, IL‐6), macrophage inflammatory protein‐1 alpha (MIP‐1 α) and hemoglobin (HB) (Zhang et al. 2021; Blanco‐Pintos et al. 2023; Cafiero et al. 2021). However, potential metabolic biomarkers have been identified from saliva and gingival crevicular fluid (GCF) samples but have not been validated so far (Antezack et al. 2020; Nguyen et al. 2020). For example, butyrate, a short‐chain fatty acid (SCFA), has previously been identified in higher concentrations in the saliva of periodontitis patients, compared to periodontally healthy patients, a finding consistent to those of studies linking SCFA production to specific periodontal pathogens such as *Porphyromonas gingivalis* and *Fusobacterium Nucleatum* (Barnes et al. 2011; Nguyen et al. 2020; García‐Villaescusa et al. 2018; Rzeznik et al. 2017; Sakanaka et al. 2022; Murakami et al. 2022; Sato et al. 2016). Lysine is an essential amino acid that contributes to periodontal epitheliums' cell renewal. Dysbiotic bacteria such as *Eikenella corrodens* secrete enzymes which convert lysine to cadaverine, depleting the lysine available for epithelial cells and producing an environment favorable to further biofilm development (Rashid et al. 2024; Levine and Lohinai 2021). Cadaverine has been linked to periodontal tissue destruction in previous metabolomic studies (Rashid et al. 2024; Kuboniwa et al. 2016; Sakanaka et al. 2017).

The aim of this review was to assess the most recent studies available on untargeted metabolomics used for periodontitis diagnosis, using saliva and GCF samples. The main focus was to provide an overview of available literature, given the relative scarcity of both studies and systematic reviews. Secondary objectives were to identify recurrent metabolites, provide an analysis of studies, and aid future research development and metabolic biomarkers' discovery.

## 2 Methods

This review complies with PRISMA 2020 reporting guidelines (Page et al. 2021). A protocol was registered at PROSPERO (CRD42023470339). This review was designed to answer the question:

Can untargeted metabolic profiling of saliva or GCF samples be used as a diagnosis tool to discern between periodontal health and periodontitis?

### 2.1 Inclusion and Exclusion Criteria

The inclusion criteria were organized according to the PECOS strategy.

P (population) = adult patients in systemic good health.
E (exposure) = patients diagnosed with periodontitis, using measurable periodontal parameters (probing depth [PD], clinical attachment loss [CAL], bleeding on probing [BOP] or other relevant periodontal indexes) (Papapanou et al. 2018).
C (control) = subjects with periodontal health (Lang and Bartold 2018).
O (outcome measures) = differences in detectable metabolites in saliva/GCF, assessed by untargeted metabolomics.
S (study types) = original studies on humans, observational and interventional designs, both retrospective and prospective.

Additional criteria:

– Studies published in the last 10 years before search conclusion (May 1, 2013, to May 1, 2023) and up to March 15, 2024 (date of actualization search) and in English.

The following exclusion criteria were applied:

– Targeted metabolomics studies.
– Studies investigating populations with specific pathologies (e.g., diabetes).
– Studies targeting pediatric populations, animal model studies and in vitro studies.
– Study designs: literature reviews and meta‐analyses, case reports, editorials, conference abstracts.
– Studies unavailable full‐text, studies with missing or incomplete data on periodontal parameters.

### 2.2 Search Strategy

An electronic search strategy was developed and conducted independently by C.A.M. and L.P.O. in three databases: PubMed, Scopus, and Web of Science. This included identifying relevant search terms, keyword‐related term branching, exploratory literature searches and database‐controlled vocabulary translation, and accommodating searching particularities of each database. The search was restricted (May 5, 2013 to May 1, 2023), to find the most recent articles available and to ensure information actuality. The process was reiterated on March 15, 2024, to discover new potentially relevant articles. The search consisted of keywords (such as Medical Subject Headings or MeSH) for metabolomics, periodontitis, and the term “diagnosis,” combined with Boolean operators “AND” and “OR,” as well as keyword searching of title, abstract and text words. Restrictions regarding language (English) and species (humans) were applied. The exact terminology used in PubMed was

(Periodontitis[tw] OR “chronic periodontitis”[tw] OR “periodontal disease*”[tw] OR “periodontal index”[tw] OR gingivitis[tw] OR “oral health”[tw] OR “periodontitis”[MeSH Terms] OR “Periodontal diseases” [MeSH Terms] OR “Periodontal Pocket” [MeSH Terms] OR “Periodontal index” [MeSH Terms] OR “gingivitis”[MeSH Terms] OR “oral health”[MeSH Terms]) AND (Metabolom*[tw] OR metabonom*[tw] OR metabolite*[tw] OR “high‐performance liquid chromatography”[tw] OR “mass spectrometry”[tw] OR “nuclear magnetic resonance spectroscopy”[tw] OR “metabolome”[MeSH Terms] OR “metabolomics”[MeSH Terms]) AND (Diagnos*[tw] OR “early diagnosis”[tw] OR “oral diagnosis”[tw] OR “diagnosis”[MeSH Terms] OR “early diagnosis”[MeSH Terms] OR “diagnosis, oral”[MeSH Terms]) Filters: Humans, English, from May 1, 2013 to March 15, 2024.

### 2.3 Study Selection Process

The results were centralized, and duplicates were identified using reference manager software Mendeley (2.84.0© 2023 Mendeley Ltd.). Studies were selected based on potential relevancy, by independent screening of title and abstract conducted by two researchers (C.A.M. and K.A.I.). All the studies considered relevant were retrieved full‐text and read by the same two researchers. Any disagreements were discussed, and a third researcher (C.D.C.) was consulted for conflict resolution. The level of agreement between researchers was established by calculating Cohen's Kappa coefficient.

### 2.4 Data Extraction

Data was extracted using a dedicated extraction form, including information on:

– General aspects (study title, first author, geographic area, publication year, study design, sample type).
– Population (number of participants, age/gender distribution, inclusion/exclusion criteria, smoking status).
– Exposure/Controls (case and control definitions, periodontal parameters, instruments used, number of examiners).
– Results/Outcomes (statistically significant metabolites, upregulated/downregulated metabolites, analytic platform employed).
– Metabolomics (type of technology, pre‐sampling procedures, sample collection, pre‐analytical procedures).

For studies comparing periodontal statuses of systemically healthy individuals with those of diseased individuals, only data corresponding to systemically healthy individuals was extracted. Studies with interventional designs were included, but only pre‐intervention metabolic data was extracted. Studies analyzing multiple sample types were included and only data corresponding to saliva or GCF samples was extracted. These measures were taken to avoid confounding.

### 2.5 Risk of Bias/Quality Assessment

Risk of bias/quality assessment was conducted independently by two researchers (N.M. and B.S.D.), using a version of the Newcastle‐Ottawa Scale (NOS) (Wells et al. 2014) modified for cross‐sectional studies as per Modesti et al. (2016). A third researcher (L.P.O.) was asked to intervene for additional assessment and conflict resolution, when necessary. One section of the scale (non‐respondents – regarding response rates) was eliminated, due to not being applicable to included studies. Ascertainment of exposure was graded one point for existing case definition criteria and two points for validated, widely accepted criteria. Confounder control was graded one point for smoking and two points for additional confounders (other oral pathologies, sample contamination, etc.). The assessment of outcome was graded one point if assessment methods were provided and two points for statistical validation. Studies with NOS scores 0–3 are considered as low quality, scores 4–6 moderate quality and scores 7–9 high quality (Luchini et al. 2017).

### 2.6 Pathway Enrichment Analysis

Statistically significant metabolites recurring in two or more studies were identified and listed for analysis. The Human Metabolome Database (HMDB) was used for identification before pathway analysis (Wishart et al. 2022). Pathway analysis was run through MetaboAnalyst version 5.0 (www.metaboanalyst.ca) (Lu et al. 2023). The *Homo sapiens* KEGG (Kyoto Encyclopaedia of Genes and Genomes) was chosen as a reference library. A hypergeometric test was chosen as algorithmic parameter and relative‐betweenness centrality was chosen for topology analysis. For a pathway to be considered significantly enriched, a less than 0.05 adjusted *p* value (FDR – false discovery rate) was considered necessary.

## 3 Results

### 3.1 Study Selection

**Figure 1:**
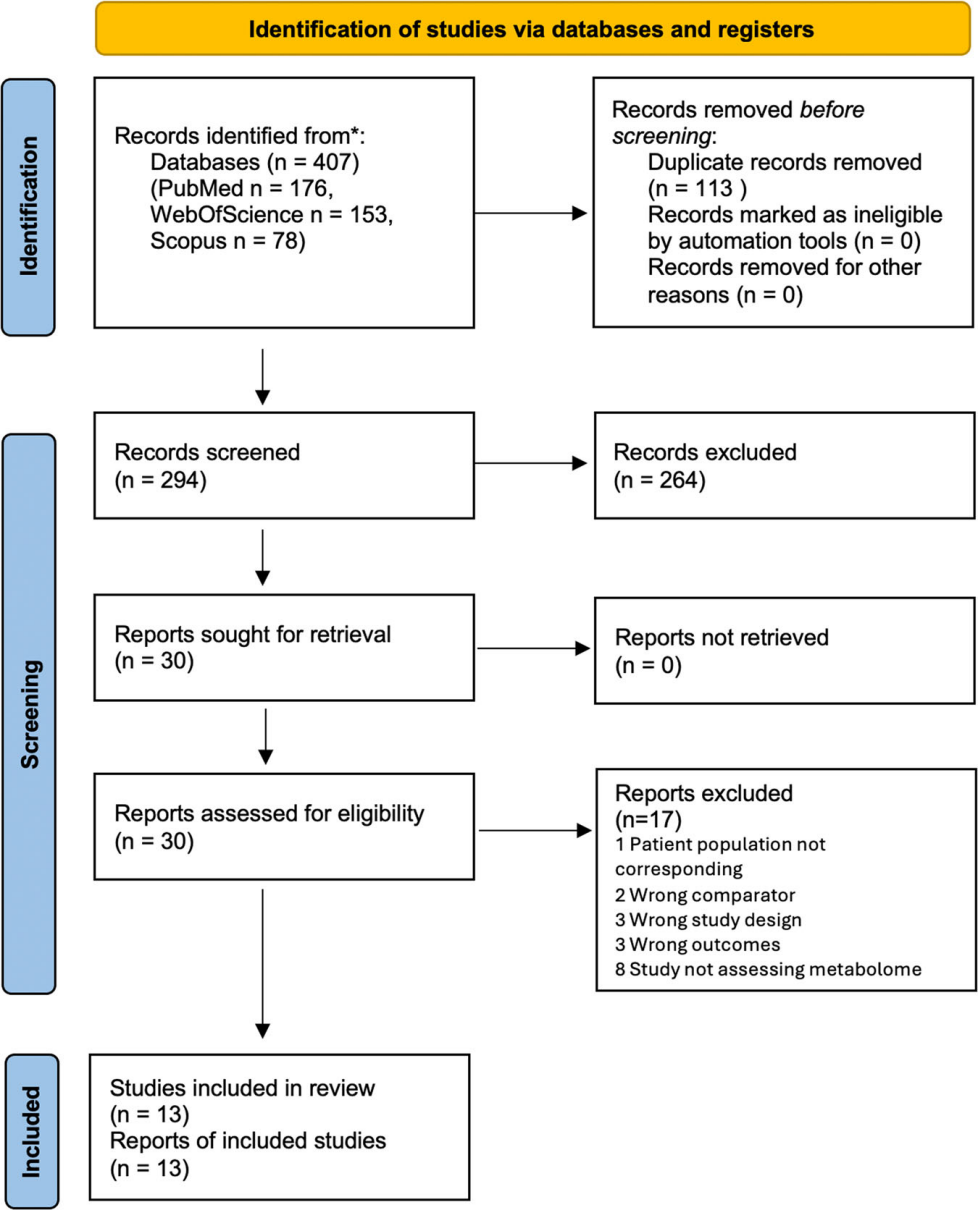
Prisma flowchart.

Out of 407 results, 30 were selected for retrieval and full‐text assessment. 17 articles were excluded (reasons detailed in Table S1) and 13 articles were included. The coefficient Cohen's kappa for inter‐researcher agreement was 0.91. Study selection progress is detailed in a Prisma flowchart (Figure 1).

### 3.2 Description of Included Studies

The studies were described by the type of sample analyzed (saliva or GCF). The general details, results and conclusions are summarized in Tables 1 and 2. Data on population characteristics, periodontal protocols and metabolomic protocols is further detailed in Tables S2–S4. Two types of labels were attributed to missing information: NA (not applicable) and NR (not reported).

**Table 1:** General characteristics/conclusions of studies using saliva samples.

Authors, Year published, Publisher	Geographic Area	Study type	Population size (analyzed)	Sample type	Diagnostic platform used	Statistically significant metabolites (nr.)	Upregulated metabolites	Downregulated metabolites	Potential biomarkers suggested	Conclusions	Notes
Barnes et al. (2014)	USA	Cross‐sectional	81 (Systemically healthy)	Saliva (unstimulated)	GC/MS LC/MS	64 of 370	P vs. H: serine, threonine, *n*‐acetylthreonine, glutamine, cadaverine, *p*‐cresol sulfate, phenylacetylglutamine, 3‐indoxyl sulfate, cysteine, cystine, *N*‐acetylmethionine, 2‐hydroxybutyrate, spermidine, cysteine‐glutathione disulfide, glycylisoleucine, glycylleucine, glutamine‐glutamate, phenylalanylvaline, leucylserine, threonylleucine, tyrosylleucine, gamma‐glutamylisoleucine, gamma‐glutamylphenylalanin, fructose, maltose, mannitol, sorbitol, glucose‐6‐phosphate, glucose, fructose‐6‐phosphate, succinate, malate, linoleate, linolenate, docosapentaenoate, oleate, dihomo‐linoleate, mead acid, arachidonate, 2‐hydroxypalmitate, butyrylcarnitine, 3‐dehydrocarnitine, acetylcarnitine, phosphoethanolamine, palmitoyl sphingomyelin, cortisol, hypoxanthine, AMP, 3′‐CMP, cytidine diphosphate, heme, adenosine 5′diphosphoribose, citalopram, cotinine.	P vs. H: tryptophylserine	NA	Untargeted salivary metabolomics can discern between healthy, gingivitis and periodontitis subjects, both with or without diabetes. Diabetic subjects showed altered salivary carbohydrate, lipid and oxidative stress metabolic signatures.	Only data from systemically healthy patients was extracted.
Bregy et al. (2019)	Switzerland	Controlled clinical trial	24	Saliva (unstimulated)	SESI‐MS	31	P vs. H: 2‐hydroxyoctanoic and hydroxynonaoic acid, pyridine, Hydroxydodecapentaenoic acid, arecoline	NA	NA	SESI‐MS metabolomics is a potential diagnosis tool for periodontitis, quick and cost‐efficient. It can also be used for monitoring therapy response.	Only data gathered before the intervention (NSPT) was considered for extraction.
Citterio et al. (2020)	Italy	Controlled clinical trial	23	Saliva (unstimulated)	^1^ H‐NMR	31	P vs. H: valine, leucine, phenylalanine, isoleucine, lactate, hypoxanthine, uracil	P vs. H: acetate, ethanol, acetoin, succinate, choline, isopropanol.	NA	^1^ H‐NMR metabolomics revealed that periodontitis patients have a different metabolic profile compared to healthy individuals, which is maintained after therapy. It could be used to differentiate between healthy and periodontitis patients and to monitor treatment.	Only data gathered before the intervention (NSPT) was considered for extraction.
García‐Villaescusa et al. (2018)	Spain	Case–control	130	Saliva (unstimulated)	^1^ H‐NMR	12 of 68	P vs. H (gr. 3 vs. 1): caproate, isocaproate+butyrate, isovalerate, isoleucine, isopropanol+methanol, 4 aminobutyrate, choline, sucrose, sucrose‐glucose‐lysine, lactate‐proline, lactate and proline.	P vs. H (gr. 3 vs. 1): Sucrose; Sucrose‐glucose‐lysine; Lactate; Proline G vs. H (gr. 2 vs. 1): Caproate; Isopropanol‐methanol; Choline	Caproate, isocaproate‐butyrate, isovalerate, lacatate+proline, proline.	No association was found between GBL and periodontitis, but ^1^ H‐NMR metabolomics discerned between periodontally healthy and periodontally affected groups, suggesting that saliva analysis could be an easy, fast, and noninvasive method to follow‐up on different diseases.	Periodontal and metabolic data extracted for whole study population.
Gawron et al. (2019)	Poland	Cross‐sectional	45	Saliva – mouth washout	^1^ H‐NMR	5 of 25	P vs. H: lactate	P vs. H: acetone, methanol, unknown 2	Acetate, acetone, isopropanol, glycerol and methanol	Metabolomics is a potentially useful approach for monitoring periodontitis. ^1^ H‐NMR analysis identified 5 potential biomarkers for CPD.	
Kim et al. (2021)	South Korea	Two‐stage cohort	271	Saliva (stimulated)	^1^ H‐NMR	19 of 31	P vs. H: taurine, isovalerate, butyrate, glucose	P vs. H: ethanol	Ethanol, taurine, isovalerate, butyrate, glucose	^1^ H‐NMR metabolic profiling of saliva of healthy patients compared to periodontitis patients brought forward 5 metabolite biomarkers for P. These biomarkers can be used for detection, screening and monitoring P. There were no significant differences between stage II and III P.	Validation cohort data not extracted.
Kuboniwa et al. (2016)	Japan	Cross‐sectional	19	Saliva (unstimulated)	GC‐MS	8 of 63	NA	Pre vs. post debridement: cadaverine, ornithine, spermidine, 5‐oxoproline	Cadaverine, 5‐oxoproline, and histidine (combination)	Post‐debridement saliva showed improved predictive performance for metabolite biomarkers discovery. PISA is useful in salivary metabolomics research.	
Na et al. (2021)	South Korea	Cross‐sectional	112	Saliva (stimulated)	^1^ H‐NMR	28	P1 vs. H: butyrate, choline P2 vs. H: acetate, butyrate, choline, propionate	PG1 vs. H: acetone, histidine, lysine, sn‐Glycero‐3‐p, pyruvate	NA	Oral metabolomic analysis discerned between healthy and periodontitis patients. The metabolome and microbiome showed a close interrelation. Multi‐omics approaches may serve as diagnostic biomarkers.	
Romano et al. (2018)	Italy	Cross‐sectional	100	Saliva (unstimulated)	^1^ H‐NMR	22	GCP vs. H: proline, phenylalanine, isoleucine, valine tyrosine GAgP vs. H: formate, phenylalanine, tyrosine	GCP vs. HI: pyruvate, *N*‐acetyl groups, lactate GAgP vs. HI: pyruvate, *N*‐acetyl groups, lactate, sarcosine	NR	GCP and GAgP seem to share the same metabolic space. ^1^ H‐NMR metabolomics analysis was able to discern both between healthy and GP or GAgP patients, thus suggesting metabolomic profiling might be a method of assessing molecular biomarkers for periodontitis diagnosis.	
Rzeznik et al. (2017)	France	Cross‐sectional	51	Saliva (stimulated)	^1^ H‐NMR	11	P vs. H: butyrate (short chain fatty acids)	P vs. H: fucose, lactate, acetate, *N*‐acetyl, gamma‐aminobutyrate (GABA), 3‐d‐hydroxybutyrate, pyruvate, methanol, threonine, ethanol	lactate, GABA and butyrate (combination)	^1^ H‐NMR metabolic profiling of saliva can differentiate patients with periodontitis from controls. This simple and minimally invasive method could be helpful in early diagnosis and follow‐up of periodontitis.	

**Table 2:** General characteristics/conclusions of studies using GCF samples.

Authors, Year published, Publisher	Geographic Area	Study type	Population size (analyzed)	Sample type	Diagnostic platform used	Statistically significant metabolites (nr.)	Upregulated metabolites	Downregulated metabolites	Potential biomarkers suggested	Conclusions
Chen et al. (2018)	China	Cross‐sectional	40	GCF	GC‐MS	20 of 349	GAgP vs. H: noradrenaline, ribose, dehydroascorbic acid, lysine, xanthine	GAgP vs. H: thymidine, glutathione, 2‐ketobutyric acid, glycine‐d5	NA	GC‐MS metabolomics can differentiate between healthy individuals and individuals with GAgP, by GCF samples. It is a noninvasive and effective method but insufficient for early diagnosis when used as a sole method.
Pei et al. (2020)	China	Cross‐sectional	58	GCF	GC‐MS	17 of 147	P vs. H: glycine‐d5, N‐carboxyglutamate 2, fructose 1, citramalic acid, 2‐butyune‐1,4‐diol, 4‐ hydroxyphenyl acetic acid, *N*‐acetyl‐β‐d‐mannosamine 1, 5‐dihydrocortisol 3, uracil, fructose 1 and N‐carbamylglutamate 2	P vs. H: lactamide 2, O‐phosphoserine 1, 1‐monopalmitin methylmalonic acid, Thymidine 3	Citramalic acid, *N*‐carbamyl‐glutamate (combination)	Differential metabolites in GCF from periodontitis patients, assessed by GC‐MS, could become biomarkers useful in diagnosis, prediction, prognosis and the management of individualized periodontal care.
Rodrigues et al. (2021)	Brazil	Cross‐sectional	120	GCF	GC‐MS	15 of 64	P vs. H: 2,3‐dihydroxypropyl inosinate, glycerol, serine, 5‐aminovaleric acid, putrescine H vs. P: lactulose, oxalic acid, 1‐benzoyl‐2‐t‐butyl‐5‐ethyl‐3‐methyl‐5‐vinyl‐ imidazolidin‐4‐one, maltose	NR	5‐aminovaleric acid, serine, 3TMS derivative	Metabolites present in the GCF of older adults, assessed by GC‐MS, could be used as biomarkers for periodontal disease detection and diagnosis.

#### 3.2.1 Studies Using Saliva Samples

Table 1 summarizes studies using saliva samples. From 10 studies, three used stimulated saliva (Rzeznik et al. 2017; Na et al. 2021; Kim et al. 2021), six used unstimulated saliva (García‐Villaescusa et al. 2018; Kuboniwa et al. 2016; Barnes et al. 2014; Bregy et al. 2019; Citterio et al. 2020; Romano et al. 2018) and one used mouth washouts (Gawron et al. 2019). One study was conducted in the United States (Barnes et al. 2014), Switzerland (Bregy et al. 2019), Spain (García‐Villaescusa et al. 2018), Poland (Gawron et al. 2019), Japan (Kuboniwa et al. 2016), and France (Rzeznik et al. 2017) each, while two studies were conducted in Italy (Citterio et al. 2020; Romano et al. 2018) and South Korea (Na et al. 2021; Kim et al. 2021) each. Total sample sizes ranged from 19 to 271 subjects. Most studies were cross‐sectional, excepting two controlled clinical trials (Bregy et al. 2019; Citterio et al. 2020), one cohort study (Kim et al. 2021), and one case–control study (García‐Villaescusa et al. 2018).

The number of identified metabolites ranged between 11 and 370, while statistically relevant metabolites ranged between 5 and 68. Potential biomarkers were suggested in five studies (García‐Villaescusa et al. 2018; Rzeznik et al. 2017; Kuboniwa et al. 2016; Kim et al. 2021; Gawron et al. 2019), from which two suggested a biomarker panel (Rzeznik et al. 2017; Kuboniwa et al. 2016). The most frequently mentioned molecule was butyrate, found upregulated in five studies (García‐Villaescusa et al. 2018; Rzeznik et al. 2017; Na et al. 2021; Kim et al. 2021; Barnes et al. 2014) and suggested as biomarker in three studies: by itself (García‐Villaescusa et al. 2018; Kim et al. 2021) and part of a biomarker panel with lactate (Rzeznik et al. 2017). Lactate was mentioned in five studies as well (García‐Villaescusa et al. 2018; Rzeznik et al. 2017; Citterio et al. 2020; Romano et al. 2018; Gawron et al. 2019) and suggested as a biomarker in one panel (Rzeznik et al. 2017). It appeared downregulated in two studies (Rzeznik et al. 2017; Romano et al. 2018) and upregulated in the other three. Isoleucine recurred upregulated in four studies (García‐Villaescusa et al. 2018; Barnes et al. 2014; Citterio et al. 2020; Romano et al. 2018). Several molecules were each mentioned in three studies, with same direction of regulation across studies: glucose (García‐Villaescusa et al. 2018; Kim et al. 2021; Barnes et al. 2014), pyruvate (Rzeznik et al. 2017; Na et al. 2021; Romano et al. 2018), proline (García‐Villaescusa et al. 2018; Kuboniwa et al. 2016; Romano et al. 2018), valine and phenylalanine (Barnes et al. 2014; Citterio et al. 2020; Romano et al. 2018), and ethanol (Rzeznik et al. 2017; Kim et al. 2021; Citterio et al. 2020). N‐acetyl groups appeared in three studies (Rzeznik et al. 2017; Barnes et al. 2014; Romano et al. 2018) but were found either upregulated (Barnes et al. 2014) or downregulated. Choline (García‐Villaescusa et al. 2018; Na et al. 2021; Citterio et al. 2020) and acetate (Rzeznik et al. 2017; Na et al. 2021; Citterio et al. 2020) were both found upregulated, excepting in one study (Citterio et al. 2020). Other molecules were each mentioned in two studies: isovalerate (García‐Villaescusa et al. 2018; Kim et al. 2021), cadaverine (Kuboniwa et al. 2016; Barnes et al. 2014), hypoxanthine/xanthine (Barnes et al. 2014; Citterio et al. 2020), spermidine (Kuboniwa et al. 2016; Barnes et al. 2014), methanol (García‐Villaescusa et al. 2018; Rzeznik et al. 2017), acetone (Na et al. 2021; Gawron et al. 2019) ‐ with same direction of regulation; and isopropanol (50, 54), threonine and hydroxybutyrate (Rzeznik et al. 2017; Barnes et al. 2014), succinate (Barnes et al. 2014; Citterio et al. 2020) – either up‐ or downregulated.

Population characteristics are included in the Table S2. Smokers were included in study populations in four studies (García‐Villaescusa et al. 2018; Rzeznik et al. 2017; Bregy et al. 2019; Romano et al. 2018), of which one distributed smokers equally among groups (Romano et al. 2018) and one did not report smokers' number or distribution (García‐Villaescusa et al. 2018). Several criteria related to possible confounding factors were considered for participants' exclusion (besides standard criteria such as pregnancy, systemic diseases, edentulism, etc.): oral conditions of the soft or hard tissues (Kuboniwa et al. 2016; Na et al. 2021; Kim et al. 2021; Barnes et al. 2014; Romano et al. 2018; Gawron et al. 2019), previous periodontal treatment (Na et al. 2021; Kim et al. 2021; Barnes et al. 2014; Citterio et al. 2020; Romano et al. 2018), salivary diseases or impaired salivary function (Kuboniwa et al. 2016; Barnes et al. 2014; Romano et al. 2018), previous use of antibiotics all but one study (Bregy et al. 2019), previous use of antimicrobial medication (Barnes et al. 2014; Bregy et al. 2019; Gawron et al. 2019), usage of anti‐inflammatory medication (Na et al. 2021; Kim et al. 2021), usage of prescription medications or medications with confirmed side effects on periodontal tissues (Kuboniwa et al. 2016; Citterio et al. 2020), and regular alcohol consumption (Rzeznik et al. 2017; Romano et al. 2018).

Periodontal assessment protocols are detailed in Table S3. The diagnostic criteria used for periodontitis assessment were the diagnosis criteria for generalized aggressive periodontitis (GAgP) (Citterio et al. 2020; Romano et al. 2018), the CDC‐AAP (Eke et al. 2012) criteria (García‐Villaescusa et al. 2018; Rzeznik et al. 2017; Kuboniwa et al. 2016), the 1999 classification (Armitage 1999) of periodontal diseases criteria (Gawron et al. 2019), and the 2017 classification of periodontal diseases (Papapanou et al. 2018) diagnostic criteria (Kim et al. 2021). Two studies used other criteria to define periodontitis (Barnes et al. 2014; Bregy et al. 2019). All but three studies (Rzeznik et al. 2017; Kuboniwa et al. 2016; Gawron et al. 2019) reported criteria to define control cases.

Sampling and pre‐sampling procedures are summarized in Table S4. All studies enforced pre‐sampling restrictions, between 1 h and a night previous. All studies attempted control of exogenous metabolite confounding by imposing restrictions on food and/or beverage consumption, excepting one study which only restricted usage of oral hygiene products (Kuboniwa et al. 2016). The following analytic platforms were used: SESI‐MS (Bregy et al. 2019), GC‐MS (Kuboniwa et al. 2016), GC‐MS and LC‐MS (Barnes et al. 2014) and ^1^H‐NMR (García‐Villaescusa et al. 2018; Rzeznik et al. 2017; Na et al. 2021; Kim et al. 2021; Citterio et al. 2020; Romano et al. 2018; Gawron et al. 2019). MVA was performed in all studies but one (Barnes et al. 2014).

#### 3.2.2 Studies Using GCF Samples

Table 2 summarizes the studies using GCF samples. Out of three included studies (Chen et al. 2018; Pei et al. 2020; Rodrigues et al. 2021), two were published in China (Chen et al. 2018; Pei et al. 2020) and one in Brazil (Rodrigues et al. 2021). All studies were cross‐sectional. Total sample sizes ranged from 40 to 120 subjects.

The number of identified metabolites ranged between 64 and 349 and statistically significant metabolites ranged between 15 and 20. Potential biomarkers were suggested in two studies, both suggesting a biomarker panel (Pei et al. 2020; Rodrigues et al. 2021). Only one metabolite was recurrent glycine‐d5, found either upregulated (Pei et al. 2020) or downregulated (Chen et al. 2018).

Population characteristics are found in Table S2. One study matched periodontitis groups and control groups in both gender and age (Chen et al. 2018). There were no smokers included. Several factors related to possible confounding recurred as exclusion criteria: previous periodontal treatment (Chen et al. 2018; Pei et al. 2020), previous orthodontic treatment (Chen et al. 2018; Pei et al. 2020), previous use of antibiotics in all studies, previous use of anti‐inflammatory medication (Chen et al. 2018), oral contraceptives use (Chen et al. 2018; Pei et al. 2020).

Periodontal assessment protocols are detailed in Table S3. Criteria used for periodontitis diagnosis were generalized aggressive periodontitis (GAgP) diagnosis (Chen et al. 2018) and the 1999 classification of periodontal diseases criteria (Pei et al. 2020). One study did not report diagnosis criteria for periodontitis or controls.

Table S4 summarizes metabolomic protocols. Two studies reported site preparation procedures (Chen et al. 2018; Pei et al. 2020) and sample collection by Periopaper and Periotron (Chen et al. 2018; Rodrigues et al. 2021).

### 3.3 Risk of Bias/Quality Assessment

The NOS scale scores attributed are detailed in Table S5. The results indicate seven studies of high quality (Kuboniwa et al. 2016; Na et al. 2021; Kim et al. 2021; Citterio et al. 2020; Romano et al. 2018; Chen et al. 2018; Pei et al. 2020), five studies of medium quality (García‐Villaescusa et al. 2018; Rzeznik et al. 2017; Barnes et al. 2014; Bregy et al. 2019; Gawron et al. 2019) and one study of low quality (Rodrigues et al. 2021).

### 3.4 Pathway Enrichment Analysis

**Figure 2:**
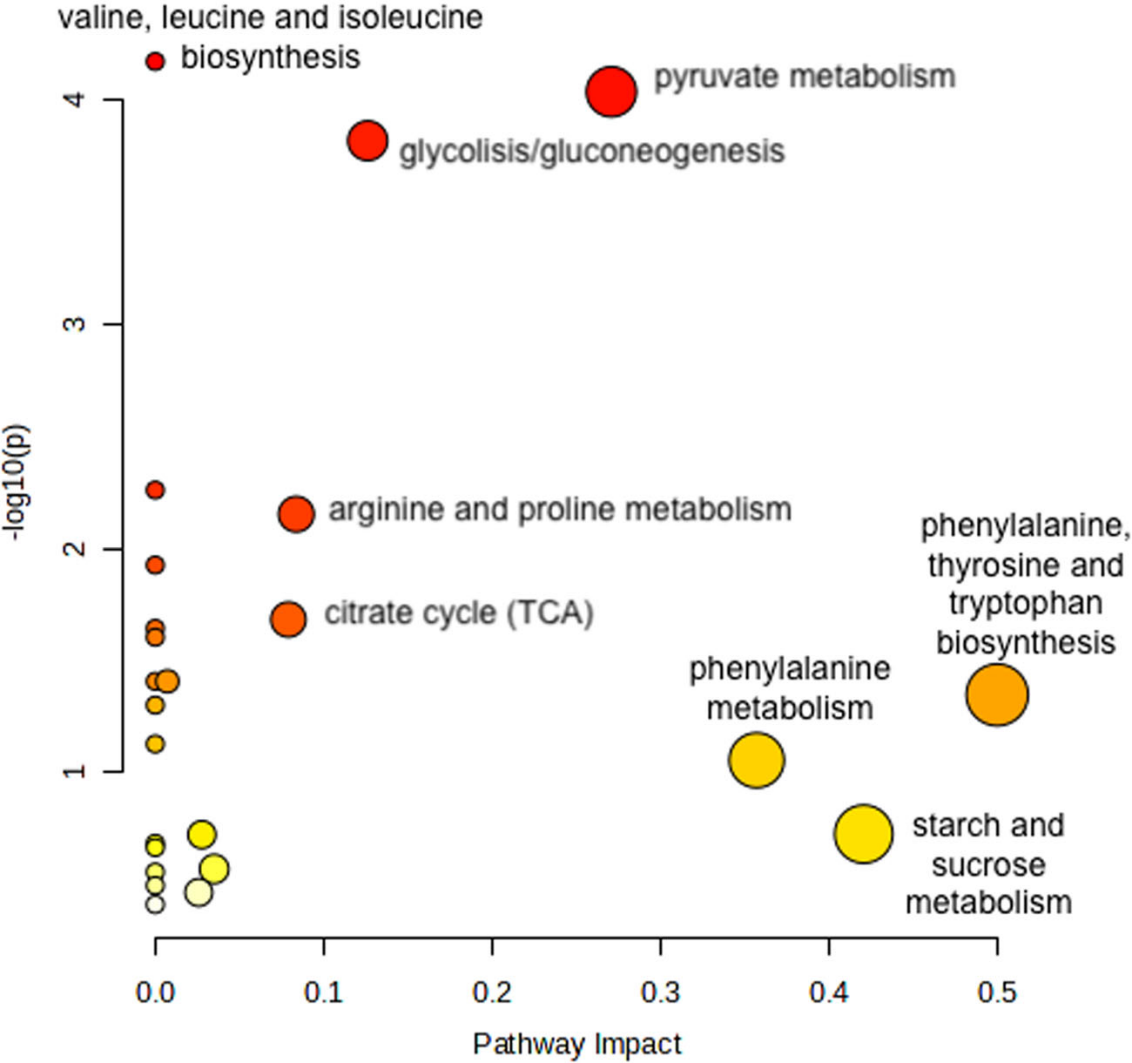
Results of the pathway analysis.

Pathway analysis was performed for the saliva‐based studies. 22 recurrent metabolites were included. Figure 2 illustrates the results. Circle size signifies the pathway impact value from topology analysis and circle color indicates significance level in the enrichment analysis. The most prominent pathways derived from the selected metabolites were valine, leucine and isoleucine biosynthesis pathway (FDR 0.0037), pyruvate metabolism (FDR 0.0037), and glycolysis/gluconeogenesis (FDR 0.004).

## 4 Discussions

This systematic review synthesized available literature from the last decade on untargeted metabolomics for periodontitis diagnosis. Study results and conclusions were analyzed separately, since saliva and GCF do not represent a single biological environment (Lynge Pedersen and Belstrøm 2019; Chen et al. 2019). 23 metabolites presented statistically significant differences between healthy and periodontally affected individuals, confirming the existence of a dysbiosis‐altered metabolic context, characteristic of periodontitis and chronic inflammation (Basic and Dahlén 2023; Mahendra et al. 2022). The metabolites belong to the amino‐acids group (isoleucine, proline, valine, phenylalanine, glycine, threonine), organic acids (lactate, pyruvate, hydroxybutyrate, succinate) and short‐chain fatty acids (SCAAs) (butyrate, isovalerate, acetate), carbohydrates (glucose), bioactive amines (cadaverine, spermidine, acetone), purines (xanthine, hypoxanthine), alcohols (methanol, ethanol, isopropanol), and other small molecules (choline). The pathway analysis identified impacted amino‐acid metabolism and energy metabolism pathways.

Regarding the various analytic platforms used for the untargeted analysis of samples, 1H‐NMR was used in most saliva‐based studies (7 out of 10), while GC‐MS was used in all 3 GCF studies. SESI‐MS was used in one saliva‐based study, while LC‐MS only appeared in conjunction with GC‐MS in one study. To clarify the impact of the choice of analytic platform on the results, we must consider the particularities of each. MS (mass spectrometry) and NMR (nuclear magnetic resonance) are the two main platform options in metabolomics (Chen et al. 2022). MS is a technique based on spectral acquisition in the form of mass‐to‐charge ratios and relative intensities of ionized compounds. It can be combined with chromatographic techniques such as liquid or gas chromatography (LC or GC), which have the role of separation, reducing the complexity of the sample and thus allowing the analysis of different molecules at different times. LC is more suited for the detection of specific types of substances, such as fatty acids, lipids, polyamines, etc. GC is limited by its ability to discern only volatile substances, or substances which can be volatilized (Patti et al. 2012; Veenstra 2012; Beisken et al. 2015). SESI (secondary electrospray ionization) is a technique that analyses trace concentrations in vapors (Choueiry and Zhu 2022). Coupling with chromatographic methods improves the discerning ability of MS, resulting in a larger number of identified compounds. However, it requires special protocols of purification or separation of samples before analysis. In comparison, NMR requires less extensive sample preparation techniques, hence providing quicker results. NMR acquires spectra by measuring the signal resulted by the resonance of protons in the molecules within a magnetic field (Nagana Gowda and Raftery 2021). While being a reproductible technique, it is less sensitive, which leads to the identification of less compounds than its counterpart. However, it can reliably identify the most abundant metabolites in a sample, thus characterizing a sample accurately (Bhinderwala et al. 2018; Emwas et al. 2013). Therefore, the impact of the analytic platforms can be observed on both the number of identified metabolites, and the nature of the identified compounds.

Changes in bacterial biofilms can indicate periodontal diseases' progression, especially when observed with combined ‐omics technologies (Basic and Dahlén 2023; Di Stefano et al. 2022; Wei et al. 2022; Belibasakis et al. 2023). Hyvärinen et al (Hyvärinen et al. 2021) state that the oral microbiome is correlated with the oral metabolome, as the metabolite content of saliva is also derived from the local microbiome. Gardner et al. (2020, 2019) reported that when compared to whole‐mouth saliva, sterile parotid gland saliva is lacking several metabolites associated with microbial activity, such as acetate, butyrate and propionate (known as short‐chain fatty acids – SCFAs). The concentrations of several salivary metabolites (SCFAs, amines, phenylalanine, succinate, glycine) were co‐related strongly with the amounts of bacteria in the saliva. This confirms the results of our review, as most identified metabolites belong to the SCFAs and amines group. SCFAs are produced from carbohydrate fermentation and during proteolytic degradation by oral and gut bacteria (Basic and Dahlén 2023). Some Gram‐negative periodontopathogens such as *P. gingivalis*, *Treponema denticola, Aggregatibacter actinomycetemcomitans, Prevotella intermedia*, and *F. nucleatum* release SCFAs as metabolic by‐products (Niederman et al. 1997; Takahashl et al. 1997, 2015). SCFA levels from GCF were found to be significantly increased in sites colonized by aforementioned bacteria and to significantly decrease after periodontal therapy (Lu et al. 2014).

A study by Liebsch et al. (2019) identified 107 salivary metabolites associated with periodontal disease, suggested to be related to tissue destruction, host defense mechanisms, and bacterial metabolism. Phenylacetae was significantly associated with periodontitis and was suggested as potential biomarker (Liebsch et al. 2019). These findings were validated Andörfer et al. (2021), who analyzed 938 salivary samples using untargeted metabolomics. Phenylacetate, other catabolists of amino acids, and N‐methylated amino acids were significantly associated with PD and with 5‐year tooth loss. Phenylacetate is a metabolite of phenylalanine and a bacterial catabolism product (Teufel et al. 2010). We identified phenylalanine as upregulated in three of the included studies, suggesting phenylalanine metabolic pathway could be involved in the progression of periodontal disease. A study by Jiang et al. (2022) concluded that phenylalanine and butyrate metabolism may contribute to periodontitis pathogenesis in patients with and without type‐2 diabetes. We also identified butyrate most frequently across included studies, along lactate. Butyrate contributes to the specific microbial ecology of periodontitis, providing a competitive advantage to some pathogens (*P. gingivalis*) by having an antimicrobial activity on some gram‐positive species. Its isomer, isobutyric acid, is produced by *P. gingivalis* and promotes *T. denticola* growth (Guan et al. 2021). High concentrations and chronic exposure to butyrate induce cytostasis and apoptosis in gingival fibroblasts, thus contributing to the progression of periodontal disease (Shirasugi et al. 2018; Kurita‐Ochiai et al. 2008). Lactate is considered a waste product of glucose anaerobic metabolism and acts as an acidogenic. A recent study suggested its role in the osteogenic differentiation of human periodontal ligament stem cells (Luo et al. 2022). Lactate dehydrogenase has been researched as a salivary marker for periodontitis, raised salivary levels suggesting tissular damage (Ansari Moghadam et al. 2022; Ali et al. 2018) and increased activity being associated with periodontal disease, the presence of calculus and deep pockets (Alonso De La Peña et al. 2007). Increased LDH levels, when associated with pyruvate metabolism pathway and possibly lowered lactate levels, could potentially indicate periodontitis.

A systematic review by Brito et al. (2022) suggested that metabolites more frequently found in individuals with periodontitis were related to both the host and to microorganism responses. The review identified phenylalanine, valine, succinate, propionate, butyrate, and acetate as frequently expressed in periodontitis, as well as the metabolic pathways involved (butanoate metabolism, pyruvate metabolism, glycolysis/gluconeogenesis, phenylalanine, tyrosine and tryptophan biosynthesis). Baima, Iaderosa, et al. (2021) identified 27 salivary metabolite biomarkers for periodontitis in a systematic review, the most frequently encountered being valine, phenylalanine, isoleucine, tyrosine and butyrate. The main impacted metabolic pathways (glycine, serine and threonine metabolism, phenylalanine metabolism, and pyruvate metabolism) are involved in bacterial energy metabolism, inflammation, immune response and oxidative stress. Our results partially overlap with aforementioned studies, suggesting that potential metabolic biomarkers could be derived from bacterial activity, rather than tissue degradation. Baima, Corana, et al. (2021) conducted another systematic review on GCF biomarkers for periodontitis and identified 10 metabolites. Malondialdehyde was upregulated in periodontitis and considered one of the most consistent markers, while glutathione was downregulated. Due to a few included studies based on GCF, we have not been able to replicate these conclusions.

The studies on GAgP (Rzeznik et al. 2017; Citterio et al. 2020; Romano et al. 2018; Chen et al. 2018) were included in this review; under the current classification of periodontal diseases, GAgP is no longer separate from periodontitis and no significant differences in metabolic profiling were identified between GAgP and chronic periodontitis (Papapanou et al. 2018; Caton et al. 2018; Tonetti et al. 2018; Rzeznik et al. 2017; Romano et al. 2018). Periodontitis was always clinically diagnosed, but on various outdated protocols, which lead to lack of uniformity and possibly errored diagnosis. Just one study used the current periodontitis diagnosis criteria, due to most studies being conducted before the implementation of current criteria. Patients suffering from gingivitis or mild periodontitis forms may have been included in control groups due to usage of unspecified probes and single examiners, thus decreasing comparability. Only three studies reported two or more examiners (Kuboniwa et al. 2016; Romano et al. 2018; Rodrigues et al. 2021), therefore interindividual agreement was unavailable for most studies. However, excluding studies or group data of patients diagnosed with gingivitis was intended to increase chances of identifying disease‐specific metabolites and excluding studies on patients with periodontitis and general pathologies favored less potential metabolic interference. Most included studies had small sample sizes, decreasing representativity. Lack of uniformity in periodontal and metabolomic protocols lead to unavailable meta‐analysis. Even if similar periodontal protocols would have been followed, usage of various metabolomic analytic platforms, sample preparation protocols and results reporting methods may have led to significant variations in results across studies. Moreover, confounding due to exogenous metabolites was a concern due to high heterogeneity of pre‐sampling and sampling procedures and the inclusion of smokers.

However, this systematic review presents some strengths. It was conducted in accordance with PRISMA guidelines for systematic reviews and PRISMA for abstracts. It followed an a priori established protocol, registered on PROSPERO. A comprehensive and specific search strategy was used, built to ensure a maximum number of search results, while also maintaining high specificity. Confounding factors were partially controlled by using detailed inclusion and exclusion criteria. This systematic review features in‐depth analysis of the protocols used in all studies, on both periodontal and metabolomic levels and represents a detailed overview of the most recent studies available in the literature while highlighting various methodology aspects to be considered in further study protocols.

## 5 Conclusions

### 5.1 Implications for Clinical Practice

Metabolomics is not a routine investigation when concerning periodontitis. There is a relative scarcity of both studies and systematic reviews on the subject and the relatively recent change in periodontal disease classification, prevented current findings from validation and clinical implementation. However, metabolomic profiling of saliva and GCF samples could become a tool to provide early diagnosis and monitor treatment efficiency and disease progression. Some metabolites could eventually be validated as biomarkers to identify dysbiotic changes in the microbiota, detecting periodontal diseases in early stages, before permanent tissular damage occurs. Ultimately, opportunities for noninvasive early diagnosis or even screening tests development might appear, leading to better treatment and a lower disease burden.

### 5.2 Implications for Research

To answer the research question, we can affirm that metabolomics can indeed represent a viable method to discern between periodontal health and periodontitis. Not only can it discern between healthy and diseased states, but it can also provide valuable insights into the metabolic context of disease, potentially revealing previously unclear connections between microbiota and host responses, from genetic to tissular level, by integrating with data obtained using other ‐omics technologies. However, metabolomics is a wide domain with many analytic possibilities available in terms of technologies used, sample types, sample processing protocols and end results. Therefore, validated metabolic biomarkers for periodontitis are still unavailable. The oral metabolome is variable and individual; therefore, future longitudinal studies are needed to validate current knowledge. However, to provide reproductible results, both implementation of current diagnosis criteria and development of standardized protocols are necessary. Further research should focus on proper assessment of periodontal status and potentially identifying metabolite shifts from one stage of disease to the next. Standardized research could provide valuable data for the validation of metabolic biomarkers in periodontal disease.

## Author Contributions

*Conceptualization:* Ana‐Maria Condor, Andreea Iuliana Kui, and Patricia‐Ondine Lucaciu. *Data curation:* Ana‐Maria Condor and Marius Negucioiu. *Formal analysis*: Andreea Iuliana Kui and Marius Negucioiu. *Funding acquisition:* Ana‐Maria Condor. *Investigation:* Ana‐Maria Condor, Daniela Cornelia Condor, and Andreea Iuliana Kui. *Methodology:* Ana‐Maria Condor, Patricia‐Ondine Lucaciu and Smaranda Dana Buduru. *Project administration:* Patricia‐Ondine Lucaciu and Smaranda Dana Buduru. *Resources:* Smaranda Dana Buduru and Marius Negucioiu. *Supervision:* Patricia‐Ondine Lucaciu. *Validation:* Andreea Iuliana Kui. *Visualization:* Ana‐Maria Condor. *Writing – original draft:* Ana‐Maria Condor, Daniela Cornelia Condor, and Andreea Iuliana Kui. *Writing – review and editing:* Ana‐Maria Condor, Andreea Iuliana Kui, and Patricia‐Ondine Lucaciu.

## Ethics Statement

The authors have nothing to report.

## Conflicts of Interest

The authors declare no conflicts of interest.

## Protocol Registration

This systematic review was registered on PROSPERO (CRD42023470339).

## Supporting information

Supporting information.

## Data Availability

The data that supports the findings of this study are available in the Supporting Information of this article.
