# Supplementary material for: Metabolomics Analysis as a Tool in Periodontitis Diagnosis: A Systematic Review
Source: Clin Exp Dent Res. 2025 Apr 2;11(1):e70095. doi: 10.1002/cre2.70095 (PMC11963077; doi:10.1002/cre2.70095)
Supplement: Supplementary file 1 — Supporting information. [file CRE2-11-e70095-s001.docx]

**SUPPLEMENTARY MATERIALS**

**List of abbreviations**

1 H-NMR = proton nuclear magnetic resonance

BOP = bleeding on probing

CAL = clinical attachment loss

CDC-AAP = Centers for Disease Control and Prevention (CDC) in collaboration with the American Academy of Periodontology

FDR = false discovery rate

FMBS = full mouth bleeding score

FMPS = full mouth plaque score

GABA = Gamma-aminobutyric acid

GAgP = generalised aggressive periodontitis

GBI = gingival bleeding index

GC/MS = gas chromatography / mass spectrometry

GCF = gingival crevicular fluid

H = healthy

LC/MS = liquid chromatography / mass spectrometry

M/F = male/female

MANOVA = multivariate analysis of variance
OPLS-DA = Orthogonal Partial Least Squares discriminant analysis

MGI = modified gingival index

MVA = multivariate analysis

NA = not applicable

NCD = non-communicable disease

NOS = Newcastle-Ottawa scale

NR = not reported

NSPT = non-surgical periodontal therapy

OPLS = Orthogonal Partial Least Squares

P = periodontitis / periodontally affected

PCA = principal component analysis

PD = probing depth

PISA = periodontal inflammation surface area

PLS-DA = Partial least squares discriminant analysis

PPD = periodontal probing depth

PSI = periodontal screening index

SCFAs = short-chain fatty acids

SESI-MS = Secondary electro-spray ionization / mass spectrometry

UHPLC/MS = ultra high-performance liquid chromatography / mass spectrometry

**Supplementary Table 1.** Reasons for article exclusion

| Author | Reason of exclusion | Details |
| --- | --- | --- |
| Andörfer et al[71] | Patient population (P) not corresponding | Part of population had known/diagnosed diabetes mellitus; subgroup analysis of systemically healthy individuals not available. |
| Antezack et al[31] | Study not assessing metabolome | Proteome profiling |
| Balci et al[82] | Study not assessing metabolome | Amino-acid profiling |
| Bellei et al[83] | Study not assessing metabolome | Proteome profiling |
| Califf et al[84] | Wrong outcome (O) | No data on differences on detectable metabolites available |
| Fornasaro et al[85] | Wrong outcome (O) | No untargeted metabolomics approaches used |
| Garcia-Contreras et al[86] | Wrong study design | In-vitro study |
| Grant et al[87] | Study not assessing metabolome | Proteome profiling |
| Haririan et al [88] | Study not assessing metabolome | Proteome profiling |
| Ngo et al[89] | Study not assessing metabolome | Proteome profiling |
| Preiano et al [90] | Wrong study design | In-vitro study |
| Sakanaka et al[91] | Wrong comparator (C) | No comparison between periodontally healthy and periodontitis patients available |
| Silva et al[92] | Wrong study design | In-vitro study |
| Singh et al[93] | Wrong comparator (C) | No comparison between periodontally healthy and periodontitis patients available |
| Surersh et al [60] | Study not assessing metabolome | Oxidative stress metabolites  Blood samples |
| Tang et al[94] | Study not assessing metabolome | Proteome profiling |
| Vernerova et al[95] | Wrong outcome (O) | No untargeted metabolomics approaches used |

**Supplementary Table 2**. Population characteristics in included studies

| **Studies using saliva samples** | | | | | | | |
| --- | --- | --- | --- | --- | --- | --- | --- |
| **Authors** | **Population size (analysed)** | **Study groups** | **Gender/age distribution** | **Smokers (%)** | **Inclusion criteria** | **Exclusion criteria** | **Study location** |
| Barnes et al.[46] | 81 systemically healthy (of 161 total) | Healthy: 25  Gingivitis (data not extracted):26  Periodontitis: 29 | Healthy group : 9M/19F, age mean 37.5 (±15.2)  Periodontitis group:  11M/15F, age mean 49.7 (±10.8) | 0% | Between 18 and 65 years, systemic health (n = 81), a minimum of 20 natural teeth (excluding third molars) | Lack of signed consent form, conditions requiring premedication before dental visits, 5 or more untreated cavities, other oral soft and hard tissue pathologies, impaired salivary function, use of antibiotics/ antimicrobial medicine 30 days prior, history of uncharacterized systemic diseases, pregnant or lactating women, participation in any other clinical study within 1 week prior, tobacco use, subjects requiring dental treatment during study, immune compromised individuals, periodontal treatment within previous month | Buffalo, New York area |
| Bregy et al.[48] | 24 | Test: 12 periodontitis patients  Control: 12 healthy patients | Test group: 5M/7F, age mean 45.6 ± 8.4  Control group: 3M/9F, age mean 37.6 ± 10.1 | Test group: 50%  Control group: 0% | ≥ 18 years; systemic health; smoking ≤ 10 cigarettes/ day | no antimicrobial agents 3 months prior, no pregnancy/ lactation. | NR |
| Citterio et al[49] | 23 | Test: 12 GP patients  Control: 11 healthy patients | Test group: 5M/7F, age mean 62 ± 4.9 years  Control group: 5M/6F, age mean 23.8 ± 0.4 | 0% | min 20 natural teeth, max 4 teeth lost due to GP, complexity factors defining the need for a complex rehabilitation not satisfied. | Smokers, periodontal treatment in previous 6 months, antibiotic use within previous 3 months, systemic conditions, use of medications with confirmed side effect on periodontal tissues, pregnant/lactating. | C.I.R. Dental School, University of Turin (Italy) |
| Garcia-Villaescusa et al.[45] | 130 | 1: 39 Periodontally healthy patients  2: 59 gingivitis/early periodontitis patients  3: 32 moderate/advanced periodontitis patients | Whole population: 50M/80F, 19 to 81 years, mean age 51.8 | NR | over 18 years | antibiotics in previous 6 months, ≥ eight teeth missing (excluding third molars), pregnant, systemic conditions | Hospital Clinico Universitario of Valencia, Spain |
| Gawron et al.[51] | 45 | Test group: 30 patients with CPD  Control: 15 periodontally healthy | Test group: 11M/19F, aged 37-68  Control group: 4M/11F, aged 23-62 | 0% | diagnosed with advanced CPD with a minimum of 20 natural teeth present adults, good general health (control group) | conditions requiring pre-medication/pre-treatment prior to dental visits/ procedures; ≥ 5 decayed untreated teeth, other diseases of hard/soft oral tissues; use of antibiotics/antimicrobial medicine 30 days prior; a history of systemic disease, pregnant/ lactating women, tobacco smokers, immune-compromised individuals. | Krakow, Malopolska area |
| Kim et al.[44] | 271 | Test group: 129 periodontitis patients  - Stage II P: 67 patients  - Stage III P: 62 patients  Control group: 92 healthy patients | Test group: - Stage II: 32M/35 F, age mean 58.34 ± 11.12  - Stage III: 38 M/24 F, age mean 56.00 ± 8.74  Control group: 44M/48F, age mean 29.93 ± 7.85 | 0% | NA | systemic diseases that might affect periodontal status, pregnancy/ breastfeeding, systemic antibiotics/anti-inflammatory drugs/ oral antiseptic agents within 6 months, periodontal therapy in last 3 months, acute infection, chronic mucosal lesions of oral cavity, less than 20 teeth, smokers, lack of signed consent form, patients with grade C periodontitis. | Department of Periodontics, Pusan National University Dental Hospital (Yangsan, Korea) |
| Kuboniwa et al.[47] | 19 | NA | 4M/15F, age mean 39.2 ± 11.6 | 0% | volunteers, employees/students, consent signed | abnormal salivary function, antibiotics usage within previous 3 months, use of prescription drugs within previous 2 weeks, past and/or present use of cigarettes, diagnosis of diseases in oral soft/hard tissues, systemic conditions. | Osaka University |
| Na et al.[43] | 112 | Test group: 79 periodontitis patients  Control group: 33 periodontally healthy | Test: 46M/33F, age mean 53.99 ± 9.55  Control: 15M/18F, age mean 30.03 ± 7.19 | 0% | not pregnant/ breastfeeding, no systemic diseases that may affect periodontal status, no antibiotics in the previous 6 months, no periodontal therapy in previous 3 months. | Usage of anti-inflammatory drugs, acute infection, chronic mucosal lesion of oral cavity, current smokers | Department of Periodontics of Pusan National University Dental Hospital, Yangsan, South Korea |
| Romano et al.[50] | 100 | GP group: 33 patients  GAgP group: 28 patients  Control group: 39 periodontally healthy patients | GP group: 63.6% M, age mean 50.5 ± 8.9  GAgP group: 64.3% M, age mean 31.1 ± 4.6  Control group: 64.1% M, age mean 46.6 ± 8.2 | GP group: 15.2%  GAgP group: 14.3%  Control: 15.4% | individuals seeking oral health consultation | less than 20 teeth, antibiotic intake within previous 3 months, periodontal treatment during previous 6 months, abnormal salivary function, diagnosis of disease in oral and hard tissues, systemic conditions that could influence periodontal status and metabolomic profile, regular alcohol consumption, pregnancy/lactation. | C.I.R. Dental School, University of Turin (Italy) |
| Rzeznik et al.[42] | 51 | Test:26 periodontitis patients  Control: 25 healthy patients | Test group: 10M/16F, age mean 42.4 ± 12.8 Control group: 9M/16F, age mean 40.7±12.4 | smokers, former smokers, non-smokers, equally distributed between groups | age range of 18–64, health insurance benefits. | no systemic diseases, no antibiotics taken during previous 3 months, no regular alcohol consumption, women not pregnant/lactating. | Bretonneau Hospital in Paris, France (AP-HP, University Paris Descartes, Paris, France) |
| **Studies using GCF samples** | | | | | | | |
| **Authors** | **Population size (analysed)** | **Study groups** | **Gender/age distribution** | **Smokers (%)** | **Inclusion criteria** | **Exclusion criteria** | **Study location** |
| Chen et al.[54] | 40 | Test: 20 GAgP patients  Control: 20 healthy patients | Test group: 9M/11F, age mean 28.4 ± 4.3 years  Control group: 10M/10F, age mean 25.7 ± 4.5 years | 0% | <35 years old, minimum of 20 natural teeth (excluding third molars), good general health | Lack of signed consent form, malocclusion, any history of systemic disease, use of antibiotics/NSAIDs 3 months prior/during study, orthodontic treatment history, periodontal treatment 3 months prior, pregnancy/lactation, use of hormonal contraceptives, smokers, immune-compromised individuals./ | Department of Periodontology, Ninth People’s Hospital Affiliated to Shanghai Jiao Tong University School of Medicine |
| Pei et al.[55] | 58 | Test group: 30 periodontitis patients  Control group: 28 periodontally healthy patients | Test: 13M/17F, age mean 39  Control: 9M/19F, age mean 35.7 | 0% | Questionnaire filled, minimum 20 natural teeth (excluding third molars) | systemic diseases, orthodontic treatment in antecedents, periodontal therapy or antibiotic usage within past 3 months, pregnant/nursing/taking hormonal contraceptives, smokers. | Department of Preventive Dentistry, Ninth People’s Hospital Affiliated with Shanghai Jiao Tong University School of Medicine |
| Rodrigues et al.[56] | 64 | Test group: 60 periodontitis patients  Control group: 60 healthy patients | Whole population: mean age 70 years, gender distribution NR | 0% | ≥65 years, non-smokers, with or without chronic periodontal disease | diabetes mellitus (controlled or not), arterial hypertension, ≤ 3 absorbent cones collected, total edentulism, antibiotic therapy during study, recent odontology intervention | NR |
| **Abbreviations:** M: male; F: female; GP: generalised periodontitis; CPD: chronic periodontal disease; GAgP: generalised aggressive periodontitis; NA: not available; NR: not reported; NSAID: non-steroidal anti-inflammatory drug | | | | | | | |

**Supplementary Table 3.** Periodontal assessment protocols in included studies

| **Studies using saliva samples** | | | | | | | | | | |
| --- | --- | --- | --- | --- | --- | --- | --- | --- | --- | --- |
| **Authors** | **Case definition** | **Control definition** | **Periodontal parameters** | | **Sites examined (nr.)** | | **Instruments used** | | **Examiners (nr.)** | |
| Barnes et al.[46] | (Periodontitis) Average full mouth MGI ≥ 2, multiple BOP sites, ≥ 2 periodontal pockets with PD ≥5 mm in ≥2 quadrants. Plaque present. | MGI ≤1.0, ≤3 BOP sites, minimal plaque present | MGI, BOP, PD, Plaque index | | 6 sites/tooth | | NR | | 1 | |
| Bregy et al.[48] | PSI 4 (PPD ≥ 6 mm) on minimum 4 sites | PSI 0, 1 or 2 in all sextants (PD ≤ 3 mm) | periodontal screening index (PSI), PD, BOP | | NR | | NR | | NR | |
| Citterio et al[49] | ≥30% of teeth with CAL ≥5 mm, presence of BOP | no interdental CAL loss in >1 non-adjacent tooth, PD ≤ 3 mm, ≤10% BoP, no radiographic evidence of bone loss | PD, CAL, presence of plaque, BoP, number and percentage of PDs ≥ 4 mm and PDs ≥ 6 mm, number of teeth, full-mouth plaque score (FMPS), full-mouth bleeding score (FMBS) | | 6 sites/tooth | | PCP UNC 15 probe (Hu-Friedy) | | 1 | |
| Garcia-Villaescusa et al.[45] | Severe P: ≥ 2 interproximal sites with ≥ 6 mm CAL (on different teeth) and ≥ 1interproximal site(s) with ≥ 5mm PD  Moderate P: ≥ 2 interproximal sites with ≥4 mm CAL (on different teeth) or ≥ 2 interproximal site(s) with ≥ 5mm PD (on different teeth)  Mild P : ≥ 2 interproximal sites with ≥ 3 mm CAL (on different teeth) and ≥ 2 interproximal site(s) with ≥ 4 mm PD or 1 site with ≥ 5mm PD  (CDC-AAP definition) | H: No sites with PPD ≥ 3 and CAL ≥ 1; FMBS < 30% (CDC-AAP definition) | PD, CAL | | 2 teeth/ quadrant, 6 sites/tooth | | Cp-12 Probe (Hu-Friedy) | | 1 | |
| Gawron et al.[51] | Advanced: PD>7 mm and CAL>5 mm  Moderate: PD 5-7 mm and CAL 3-4 mm  Mild: PD 3-5 mm and CAL 1-2 mm | NR | Approximate plaque index API, sulcus bleeding index (SBI), PD, CAL. | | 5 teeth (at least 1 central incisor and 1 first molar) for P group; 5 random teeth for H | | NR | | NR | |
| Kim et al.[44] | Stage II: interdental CAL 3–4 mm; radiographic bone loss 15% - 33% of root length; maximum PD ≤ 5 mm; no tooth loss due to periodontitis; Stage III: interdental CAL ≥5 mm; radiographic bone loss extended to mid-third of root and beyond; maximum PD ≥ 6 mm; ≤4 teeth tooth loss due to periodontitis | No detectable interdental CAL | plaque index (PI), PD, CAL, BOP | | 6 sites/ tooth | | NR | | 1 | |
| Kuboniwa et al.[47] | Severe P: ≥ 2 interproximal sites with ≥ 6 mm CAL (on different teeth) and ≥ 1interproximal site(s) with ≥ 5mm PPD  Moderate P: ≥ 2 interproximal sites with ≥4 mm CAL (on different teeth) or ≥ 2 interproximal site(s) with ≥ 5mm PPD (on different teeth)  Mild P : ≥ 2 interproximal sites with ≥ 3 mm CAL (on different teeth) and ≥ 2 interproximal site(s) with ≥ 4 mm PPD or 1 site with ≥ 5mm PPD  (CDC-AAP definition) | NR | PD, BOP, CAL, PISA | | 6 sites/tooth | | NR | | 5 | |
| Na et al.[43] | moderate-to-severe periodontal disease: PD > 5 mm, CAL > 3 mm, radiographic evidence of bone loss. | low scores of BOP in <10% of the sites, no sites with PD > 3 mm or CAL. | PD, CAL, gingival index (GI), plaque index (PI) | | NR | | NR | | 1 | |
| Romano et al.[50] | GCP: ≥30% sites with PD and CAL > 5 mm, BoP present  GagP: <35 years of age, ≥6 permanent first molars and incisors with ≥1 site with PD and CAL > 5 mm, ≥ 6 other teeth also presenting ≥1 site each with PD and CAL > 5 mm. | PD and CAL ≤ 3 mm at all sites on all teeth, no radiographic evidence of alveolar bone loss, <15% BOP. | Presence/ absence of plaque(PI), BoP, PD, CAL | | 6 sites/tooth | | PCP UNC 15 probe,, Hu-Friedy | | 2 | |
| Rzeznik et al.[42] | ≥ 2 interproximal sites with CAL ≥ 3 mm, and ≥ 2 interproximal sites with PD ≥ 4 mm (not on the same tooth) or one site with PD 5mm (CDC-AAP definition) | NR | PD, CAL, radiographically assessed alveolar bone loss, BOP, Number of Residual Teeth (NRT), Decay Missing Filled index (DMF), Plaque Control Record (PCR), Gingival Bleeding Index (GBI) | | 4 sites/ tooth | | Unspecified probe, Hu-Friedy | | 1 | |
| **Studies using GCF samples** | | | | | | | | | | |
| **Authors** | **Case definition** | **Control definition** | **Periodontal parameters** | **Sites examined (nr.)** | | **Instruments used** | | **Examiners (nr.)** | |  |
| Chen et al. [54] | CAL ≥5 mm in ≥ 2 teeth in addition to incisors and first molars (>30% examination sites involved), radiographic evidence of bone loss | PD ≤3 mm and CAL = 0 mm for all teeth | PD, CAL, radiographic assessment of bone loss | NR | | NR | | 1 | |  |
| Pei et al.[55] | ≥ 4 teeth showing minimum 1 site PD ≥ 4 mm, CAL ≥ 3 mm at same site, and presence of BOP | PD ≤ 3 mm, CAL < 1 mm for all teeth | PD, CAL, BOP | 6 sites/tooth | | NR | | 1 | |  |
| Rodrigues et al.[56] | NR | NR | CAL, PPD | 6 sites/tooth | | NR | | 2 | |  |
| **Abbreviations:** MGI: Modified Gingival Index; BOP: Bleeding On Probing; PD: Probing depth; PDs: probing depth sites; PSI: Periodontal Screening Index; CAL: Clinical attachment loss; API: Approximate Plaque Index; FMPS: full-mouth plaque score; FMBS: full-mouth bleeding score; P: periodontitis; H:health (periodontal); SBI: sulcus bleeding index; CDC-AAP: Center for Disease Control/American Association of Periodontology; PI: plaque index; GI: gingival index; PISA: periodontal inflammation surface area; CPD: chronic periodontal disease; GCP: generalised chronic periodontitis; GAgP: generalised aggressive periodontitis; NA: not available; NR: not reported. | | | | | | | | | |  |

**Supplementary Table 4.** Metabolomic pre-sampling, sampling and analysis procedures of included studies.

| **Studies using saliva samples** | | | | | | | | |
| --- | --- | --- | --- | --- | --- | --- | --- | --- |
| **Authors** | **Sample collection methods** | | | | | | **Analytic platform** | **MVA** |
|  | **Restrictions** | **Sample type** | **Volume/quantity sampled** | **Sample method** | **Time of sample collection** | **Storage** |  |  |
| Barnes et al.[46] | No eating/drinking (excluding water), no brushing (excluding previous night) | Unstimulated saliva | ≥ 0.5 ml | NR | Morning | -80° C | GC-MS, LC-MS | NR |
| Bregy et al.[48] | No eating/drinking and brushing | Unstimulated saliva | NR | NR | NR | -80° C | SESI-MS, UHPLC-MS/MS | PCA, MANOVA |
| Citterio et al[49] | no eating/drinking, rinsing with mouthwashes or toothbrushing | Unstimulated saliva | 1.0 mL | Free draining method | Between 9:00 and 11:00 a.m. on day after examination | -80° C | ^1^ H-NMR | OPLS-DA |
| Garcia-Villaescusa et al.[45] | no food, chewing gum, brushing/oral hygiene products, no smoking | Unstimulated saliva | NR | Free draining method | Morning | -80˚C | ^1^ H-NMR | PCA, PLS-DA |
| Gawron et al.[51] | no eating/ drinking (except water), no brushing (excluding previous night) | Saliva - mouth washouts | 0.5 ml | mouth washout - 0.5 ml sterile saline, rinsed for 20-30s | Morning | Frozen | ^1^ H-NMR | OPLS-DA |
| Kim et al.[44] | No food/drinks, brushing, mouth wash | Stimulated saliva | NR | Cotton roll in mouth for 1 min - Salivette | 9:00 AM and 11:00 AM. | -80 C | ^1^ H-NMR | PCA , OPLS-DA |
| Kuboniwa et al. | no brushing/ using mouthwash | Unstimulated saliva | ≥ 3 ml | NR | 1:00 and 3:00 p.m. | −80 °C | GC-MS | OPLS |
| Na et al. | No food, brushing, flossing | Stimulated saliva | NR | Cotton ball in mouth for 5 min – Salivette | NR | −80 °C | ^1^ H-NMR | OPLS-DA |
| Romano et al. | No brushing/ mouthwash | Unstimulated saliva | 1 ml | Free draining method | between 8:00 and 10:00 am, 24 hours after examination | Frozen | ^1^ H-NMR | PCA/CA |
| Rzeznik et al. | No eating/drinking, no chewing gum, no brushing | Stimulated saliva | 10 ml | paraffin wax-stimulation | 09:00 and 11:00 am | -25°C | ^1^ H-NMR | PCA, OPLS |
| **Studies using GCF samples** | | | | | | | | |
| **Authors** | **Sample collection methods** | | | | | | **Analytic platform** | **MVA** |
|  | **Collection site and site preparation** | | **Volume/quantity sampled** | **Sample method** | **Time of sample collection** | **Storage** |  |  |
| Chen et al. [54] | Collection site: PD ≥5 mm for GAgP patients; tooth air-dried, supragingival plaque removed, cotton roll isolation | | Periotron 8000 | Periopaper- 20 strips/patient, inserted for 30s | NR | -80° C | GC-MS | PCA, OPLS-DA |
| Pei et al.[55] | Collection site: PD ≥5 mm, NR | | NR | Absorbent paper points – 3/patient | NR | −80 °C | GC-MS | PLS-DA |
| Rodrigues et al.[56] | Collection site: deepest PD for every quadrant; tooth air-dried, supragingival plaque removed, cotton roll isolation | | Periotron 8000 | Periopaper- 8 strips/patient, inserted for 30s | NR | −80 °C | GC-MS | PCA, OPLS-DA |
| **Abbreviations:** GC-MS: gas chromatography coupled with mass spectrometry; MS: mass spectrometry; LC-MS: liquid chromatography coupled with mass spectrometry; SESI-MS: secondary electrospray ionization coupled to  mass spectrometry; UHPLC-MS: Ultra-high performance liquid chromatography coupled with mass spectrometry;  ^1^ H-NMR: Proton nuclear magnetic resonance; PCA: principal component analisys; MANOVA: multivariate analysis of variance; OPLS-DA: Orthogonal partial least squares discriminant analysis; PLS-DA: Partial Least-Squares Discriminant Analysis; PCA/DA: component analysis and discriminant analysis; PD: probing depth; GagP: generalised aggressive periodontitis; NA: not available; NR: not reported. | | | | | | | | |

**Supplementary Table 5.** NOS Quality assessment

| **Study** | **Selection** | | | **Comparability** | **Outcome** | | **NOS Score** |
| --- | --- | --- | --- | --- | --- | --- | --- |
|  | **Sample size adequate?** | **Cases representative?** | **Ascertainment of exposure** | **Confounder control** | **Assessment of outcome** | **Statistical test** |  |
| Barnes et al.[55] | - | - | * | ** | * | * | 5/9 |
| Bregy et al.[56] | - | - | * | * | * | * | 4/9 |
| Chen et al.[62] | - | * | ** | ** | ** | * | 8/9 |
| Citterio et al[57] | - | * | ** | ** | ** | * | 8/9 |
| Garcia-Villaescusa et al.[37] | - | * | ** | * | * | * | 6/9 |
| Gawron et al.[59] | - | - | * | ** | ** | * | 6/9 |
| Kim et al.[54] | - | * | ** | ** | ** | * | 8/9 |
| Kuboniwa et al.[45] | - | * | ** | ** | ** | * | 8/9 |
| Na et al.[53] | - | * | ** | ** | ** | * | 8/9 |
| Pei et al.[63] | - | * | ** | ** | ** | * | 8/9 |
| Rodrigues et al.[64] | - | - | - | * | * | * | 3/9 |
| Romano et al.[58] | * | * | ** | * | ** | * | 8/9 |
| Rzeznik et al.[38] | - | * | ** | * | * | * | 6/9 |
